# The mediating role of organizational commitment in the relationship between head nurses’ ambidextrous leadership and team performance

**DOI:** 10.1186/s12912-026-04368-7

**Published:** 2026-02-07

**Authors:** Ying Wang, Yu Wang, Zhuoyun Wang

**Affiliations:** 1 School of Health Management, Anhui Medical University, Hefei , 231241, China; 2 The Second Affiliated Hospital of Anhui Medical University, Hefei , 230601, China

**Keywords:** Nurses, Ambidextrous leadership, Organizational commitment, Team performance, Mediation effect

## Abstract

**Background:**

Building high-performing nursing teams is crucial for hospital competitiveness and high-quality care delivery. Although ambidextrous leadership enhances team performance, its underlying mechanisms remain underexplored. This study examines how ambidextrous leadership (integrating transformational and transactional styles) affects nursing team performance in Chinese hospitals, focusing on the mediating role of organizational commitment.

**Methods:**

This study employed a cross-sectional survey design, targeting nursing staff at a public hospital in Hefei City, Anhui Province. Data on employees’ demographic characteristics, perceptions of ambidextrous leadership styles, organizational commitment, and team performance were collected via questionnaires. Structural equation modelling (SEM) analysis was conducted using AMOS software to validate the hypothesised relationships and mediating effect pathways.

**Results:**

A total of 634 nurses participated in this study. The mean team performance score was (25.50 ± 3.65). The study revealed that team performance was positively correlated with organizational commitment (*r* = 0.527, *P* < 0.01) and perceived ambidextrous leadership (*r* = 0.487, *P* < 0.01). Organizational commitment partially mediated the relationship between head nurses’ ambidextrous leadership and team performance, with a mediation effect value of 0.027. This mediation accounted for 52.94% of the total effect.

**Conclusion:**

Ambidextrous leadership exerts a positive influence on nursing team performance, with organizational commitment acting as a partial mediator. These findings help to clarify the mechanism through which leadership affects performance in healthcare settings. In practical terms, the results suggest that nursing managers can enhance team outcomes by cultivating ambidextrous leadership behaviors and actively fostering organizational commitment among nurses.

**Clinical trial number:**

Not applicable.

## Introduction

The nursing team represents the largest group within hospital organizations, and its performance is directly linked to the hospital’s high-quality development. Research consistently shows that a head nurse’s leadership style directly impacts nursing quality and broader organizational outcomes [1]. For instance, transformational leadership in head nurses has been shown to enhance team performance [2], whereas abusive leadership undermines it [3]. In line with this, the World Health Organization’s 2020 report on the global nursing workforce underscored the vital importance of nursing leadership [4].

As the nursing workforce becomes increasingly younger and educationally advanced, nurses are placing greater emphasis on their professional development and the expression of their rights. The traditional, single leadership style is no longer adequate to meet the complex and dynamic management demands. However, current research on nursing leadership still largely focuses on single leadership styles [5]. To address these management needs, some scholars have turned their attention to a composite leadership style. Studies have found that the effects of a composite leadership style surpass those of a single leadership style [6]. Given the significant impact of nursing leadership on the internal functioning of organizations, a deeper exploration of the mechanisms underlying the composite leadership style is beneficial for improving scientific management.

Ambidextrous leadership, a more complex yet potentially more effective and adaptable leadership style compared to the traditional single leadership approach, has garnered significant attention in academic research. At its core, ambidextrous leadership represents the micro-level embodiment and critical enabler of organizational ambidexterity theory. By introducing the concept of ambidexterity to the individual level, Mom (2009) emphasized how top managers can paradoxically integrate seemingly opposing elements to achieve synergistic outcomes [7]. Rosing (2011) subsequently formalized and validated the concept of ambidextrous leadership, defining it as a behavioral dynamic in which leaders flexibly and situationally switch between opening and closing behaviors to enhance followers’ capacities for exploration and exploitation [8]. Luo et al. (2016) further conceptualized ambidextrous leadership as a dynamic process that employs paradoxical and integrative thinking to resolve underlying tensions [6].Refer to existing research findings, the core of ambidextrous leadership lies in the coordination and integration of conflicting leadership forces [9], enabling it to adapt to complex management environments and contribute to the long-term competitive advantage of organizations [10]. Due to varying research perspectives, the combinations of ambidextrous leadership are not uniform [11]. The most representative perspectives include the cognitive, power, and routine-based perspectives. Among these, ambidextrous leadership from the routine-based perspective consists of two leadership styles: transactional and transformational leadership [12]. These two leadership styles each have their own strengths and weaknesses, yet complement one another.While existing studies have demonstrated that ambidextrous leadership from the routine-based perspective positively influences team innovation performance [13], research on the impact of ambidextrous leadership on nurse team performance in the nursing field remains relatively scarce.

Organizational commitment represents the affective aspect of employee behavior and is defined by Meyer and Allen (1984) as an individual’s level of involvement, loyalty, and identification with an organization [14]. Research indicates that ambidextrous leadership has a positive impact on organizational commitment [15]. Organizational commitment is a key indicator of subordinate loyalty [16], and highly committed employees develop a strong sense of belonging and identification with the organization. Their motivation stems from the internalized alignment with organizational goals, making them more willing to actively engage and dedicate themselves to organizational tasks. These employees are also more likely to contribute their strengths to the organization, thereby creating favorable conditions for achieving high performance. Nurse leaders, by flexibly applying ambidextrous leadership and switching between different leadership styles, can enhance subordinates’ organizational commitment [17], reduce nurses’ turnover intentions and burnout, foster positive leader-member relationships [18], and stabilize staffing structures, thus providing strong support for the healthy development of the organization.

This study aims to explore the role, impact, and underlying mechanisms of ambidextrous leadership from the routine-based perspective on team performance outcomes, and to verify the mediating effect of organizational commitment. This research seeks to enrich the understanding of the influence mechanisms of ambidextrous leadership in the Chinese context and provide a reference for hospital managers in formulating effective management policies and leadership training programs for head nurses.

### Theoretical research and hypotheses

#### Leader-Member exchange theory

To investigate the mechanisms through which ambidextrous leadership influences nursing team performance, this study employs leader-member exchange (LMX) theory as its theoretical framework. Originating from social exchange theory, LMX theory conceptualizes leader-member interactions as dynamic, reciprocal exchange processes [19]. The theory posits that the quality of the relationship between leaders and lsubordinates significantly impacts both performance outcomes and job satisfaction. Research demonstrates that high-quality leader-member exchanges substantially enhance employees’ job satisfaction and organizational commitment, whereas low-quality relationships may lead to employee alienation and diminished performance [20].

**Fig. 1:**
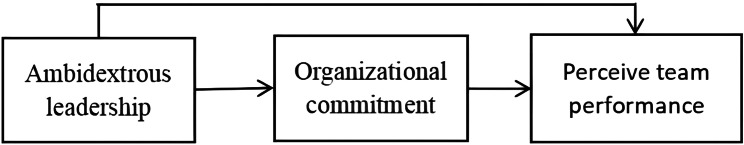
Theoretical model diagram

As an established framework in organizational behavior research, LMX theory provides valuable insights into understanding dynamic team interactions while offering practical guidance for leaders to effectively manage subordinates and enhance overall team performance. Grounded in this theoretical foundation, the present study establishes a mediation model with routine-based ambidextrous leadership as the independent variable, nursing team performance as the dependent variable, and perceived organizational commitment as the mediating variable, aiming to systematically elucidate the underlying mechanisms among these variables. (Fig. 1).

##### Hypothesis1(H1):ambidextrous leadership has a positiveeffect on team performance

With the rapid changes in the external environment, a single leadership style is no longer sufficient to meet the needs of hospital administrators. In the face of the inherent contradictions and tensions in management practices, leaders must flexibly adjust their behaviors to enhance the directional focus, dynamic transformation, and integration of their leadership actions. It is in this context that a new leadership style based on paradoxical thinking, ambidextrous leadership, has emerged [21].

Supporting evidence demonstrates that ambidextrous leadership yields significant benefits in healthcare settings. Research by Jiaqi Yan et al. (2024) indicates that during the COVID-19 pandemic, ambidextrous leadership significantly enhanced nurses’ psychological well-being by promoting work-family enrichment, which in turn led to improved patient care quality as a result of their positive psychological state [22]. Similarly, a study by Mutonyi et al. (2024) on Norwegian healthcare organizations found that ambidextrous leadership fostered innovation in healthcare services by enhancing the creative performance of healthcare workers, which played a crucial role in improving both patient experience and care quality [23]. Additional research confirms that routine-based ambidextrous leadership positively influences team innovation performance [24], with transactional and transformational leadership styles exhibiting synergistic effects that enhance team innovation.

Transformational leadership focuses on addressing subordinates’ needs, stimulating intrinsic work motivation, elevating personal aspirations to organizational levels, and aligning individual goals with organizational objectives to facilitate goal achievement [25]. Empirical studies confirm that transformational leadership promotes the construction of team effectiveness in hospitals [26], which is conducive to the improvement of the quality of medical services. Furthermore, transformational leaders can effectively improve employee performance through transformational competency training that is purposefully designed and planned [27].

In contrast, transactional leadership emphasizes task orientation, with leaders focusing on performance-contingent rewards, providing clear instructions, and establishing specific work objectives [25]. Waldman et al. (1990) posit that transformational leadership acts as a complementary force to transactional leadership, augmenting its effects to benefit both subordinates and organizations [28]. Some studies have found that transactional leadership not only has a positive effect on improving employee performance [29], but can also significantly affect team performance [30].

In synthesis, existing theoretical frameworks and empirical evidence indicate that transformational leadership revitalizes teams, while transactional leadership complements these effects to significantly enhance performance. Given the diverse needs of subordinates, neither purely material nor psychological incentives alone sufficiently address employee requirements. Ambidextrous leadership provides contextual adaptability, enabling leaders to dynamically alternate between transformational and transactional behaviors according to situational demands [31]. This synergistic integration not only improves patient experience and care quality but also establishes collaborative partnerships, fosters innovation and team performance, and implements effective governance models. This leads to the following hypothesis H1: Ambidextrous leadership style has a positive impact on team performance.

##### Hypothesis4(H4):ambidextrous leadership affects team performance through organizational commitment

Existing research on the effects of ambidextrous leadership has provided limited insights into its underlying mechanisms. Based on hypotheses H1, H2, and H3, organizational commitment emerges as a mediating variable linking ambidextrous leadership and team performance. To explore the mediating role of organizational commitment between ambidextrous leadership and team performance, we propose the following hypothesis: H4: Organizational commitment mediates the relationship between ambidextrous leadership and team performance.

##### Hypothesis2(H2):ambidextrous leadership has a positive impact on organizational commitment

A review of existing literature indicates that the relationship between transformational leadership and organizational commitment has been more extensively studied than that involving transactional leadership. Transformational leadership fosters employee trust and respect toward leaders, enhances motivation, promotes self-regulation and evaluation, and ultimately strengthens commitment to the organizational mission. Transactional leadership, being outcome-oriented and responsive to employees’ needs, can also enhance employees’ identification with the organization and thereby positively influence their organizational commitment. In a study of specialist nurses, Tao Jia et al. found that organizational commitment and perceived organizational support serially mediate the relationship between ambidextrous leadership and knowledge-sharing behavior, with ambidextrous leadership exerting a positive effect on organizational commitment [32]. Current research, both domestic and international, suggests that both transformational and transactional leadership are positively correlated with employees’ organizational commitment. From a routine perspective, the construct of ambidextrous leadership reflects the synergistic interplay of these two styles, emphasizing their complementary and concurrent application. Accordingly, the following hypothesis is proposed: H2:Ambidextrous leadership positively affects organizational commitment.

##### Hypothesis3(H3):organizational commitment has a positive effect on team performance

Substantial international research has established a significant correlation between organizational commitment and team performance, encompassing both collective outcomes and individual member contributions [33]– [34].Empirical evidence specifically from healthcare contexts demonstrates this relationship, as Baird et al. confirmed that organizational commitment positively influences the performance of hospital health personnel [35]. Enhanced organizational commitment among employees leads to improved individual job performance, which subsequently elevates overall team performance. Therefore, we propose the following hypothesis: H3: Organizational commitment has a positive effect on team performance.

### Objects and methods

#### Research objects

This study utilized a cross-sectional design with convenience sampling. From April to July 2025,706 clinical nurses were recruited from a provincial tertiary A-grade hospital in Anhui Province. The sample hospital is a comprehensive medical institution with approximately 200,000 square meters of floor space, 2,600 operational beds, an annual outpatient volume exceeding 2 million visits, and approximately 110,000 annual discharges. The hospital employs over 3,000 staff members, including more than 600 senior-level professionals. As a regional medical center integrating healthcare, teaching, and research functions, it provides representative data for this investigation.

Inclusion criteria: formal employees of the hospital with a nursing license; nurses on duty who have been engaged in clinical nursing in the hospital for ≥ 1 year; nurses who gave informed consent and voluntarily participated in this surve. Exclusion criteria: unofficial employees; rotating staff, trainees, and intern nurses; and those who have been transferred within their work units during the survey period.

The study received ethical approval from the hospital ethics committee (Approval No: YX2025-206).

#### Sample size and sampling

Kendall’s sample size estimation method [36], which requires a sample size 5 to 10 times the number of variables, was employed. With 44 items in this study, the minimum sample size was 264, meeting the minimum requirement for ensuring structural model stability [37]. Accounting for a potential 10% to 20% non-response rate, the final sample size was determined to be 330 participants. A total of 706 nurses completed and returned the questionnaire. Seventy-two questionnaires were excluded due to non-compliance with inclusion criteria, completion time under three minutes, or non-standardised responses. Ultimately, 634 valid questionnaires were included, yielding an effective response rate of 89.80%.

## Methods

### Research instrument

#### General information questionnaire

Designed by the researcher, it contains age, gender, marital status, nursing age, length of time working with the head nurse, education, title, and department.

#### Ambidextrous leadership style measurement scale

This scale was adapted from the MLQ-5X developed by Bass et al. (1995) through translation and revision by Liu Z et al. (2014) [38].The scale comprises 13 items (5 for transactional and 8 for transformational leadership). It employs a 5-point Likert scale ranging from 1 (“never”) to 5 (“frequently”), based on subordinates’ perceptions of how often their leaders exhibit the described behaviors. All items are positively scored. Higher scores reflect a higher perceived frequency of the leadership behaviors. If the score for transactional leadership is higher than that for transformational leadership, nurses perceive their head nurse’s style as predominantly transactional, and vice versa.The nurses perceive a more transformational leadership style. The nurse manager’s leadership style is “more transformational than routine”.Ambidextrous leadership was quantified using the product-term approach [39], calculated by first obtaining the mean scores for each leadership style and then multiplying the two means. This method captures the synergistic effect between the two styles. The Cronbach’s alpha coefficients for the transactional and transformational leadership subscales in this study were 0.857 and 0.942, respectively.

#### Chinese workers’ organizational commitment scale

Organizational commitment was measured using the Chinese Organizational Commitment Questionnaire (COCQ) developed by Ling Wenquan et al. (2001) [40]. This instrument was designed for the Chinese context and expands upon the Western three-factor model (affective, normative, and continuance commitment) by incorporating two additional dimensions: ideal commitment and opportunity commitment. Responses were recorded on a 5-point Likert scale (1 = “strongly disagree” to 5 = “strongly agree”). The total score ranges from 25 to 125, with higher scores indicating a greater level of organizational commitment. The Cronbach’s alpha for the scale in this study was 0.952.

#### Team performance measurement scale

Team performance was assessed using a 6-item scale translated and adapted into Chinese by Chen Guoquan [41] from the original work of Barker, Tjosvold, & Andrew (1988). Respondents rated their agreement with each item based on their perception of their department (team).It employs a 5-point Likert scale (1 = “strongly disagree” to 5 = “strongly agree”), yielding a total score between 6 and 30. Higher scores indicate better team performance. In this study, the scale demonstrated excellent reliability with a Cronbach’s alpha of 0.970.

The studies all use existing mature scales at home and abroad, and each variable is measured under the perception of individual members.

### Research methods

#### Data collection methods

Following informed consent from the hospital’s governing body, a questionnaire survey was conducted via Wenshu Xing. The questionnaire header included introductory remarks, research objectives, and an informed consent form. Prior to formal implementation, 52 nurses were invited to participate in a pilot study to assess completion time and refine wording for validity. Subsequently, researchers distributed the questionnaire via a Wenshu Xing link, enabling eligible nurses to complete it anonymously and voluntarily via mobile or computer. All questions were mandatory, and each IP address could only complete the questionnaire once, ensuring completeness and preventing duplication.

#### Statistical methods

Data were analyzed using SPSS 25.0 and AMOS 26.0 software. Common method bias was examined via Harman’s single-factor method (CFA approach). Demographic variables were described using frequencies and percentages. Normality testing followed Kim (2013) [42], where a sample size > 300, skewness < 2, and kurtosis < 7 indicated approximate normality. In this study, skewness was 0.097 and kurtosis 0.194. Concurrently, QQ plots assessed data normality, confirming all continuous variables met approximate normality. Data were statistically described using means and standard deviations. Pearson correlation analysis examined relationships among ambidextrous leadership of head nurses, organizational commitment of nurses, and perceived team performance. Box plots identified eight potentially anomalous observations, which were retained after comprehensive consideration. Firstly, given the substantial sample size (*N* = 634), these outliers were anticipated to exert negligible influence on overall parameter estimates. Secondly, retrospective review of original questionnaires revealed no evidence of random responses or logical inconsistencies, suggesting these likely represent genuine respondent reactions reflecting inherent individual cognitive variations within nursing teams. Finally, to validate the robustness of this decision, we conducted key model fits excluding these outliers. Results indicated no substantive alteration to the model conclusions. The Variance Inflation Factor (VIF) values for all variables were below 5, confirming no significant multicollinearity issues among variables. In summary, by constructing a structural equation model, fitting and refining the data using the maximum likelihood ratio method, and employing the Bootstrap method to test the significance of the organizational commitment mediating effect, we conducted our analysis. The significance level was set atα = 0.05. Model fit evaluation criteria: Chi-square/degrees of freedom ratio (χ²/df) < 3 indicates excellent fit, < 5 is acceptable; root mean square error of approximation (RMSEA) < 0.080; standardised root mean square residual (SRMR) < 0.05; the Comparative Fit Index (CFI), Normative Fit Index (NFI), and Tucker–Lewis Index (TLI) must all exceed 0.900 to indicate good model fit [43].

## Results

### Test for common method bias (CMB)

The study used self-reported data, which may have bias issues. Following the recommendations of Podsakoff et al. (2003) [44], the Harman single-factor method (CFA method) was employed to examine common method bias. The CFA method considers that if the fit index of the one-way CFA model does not meet the criteria of goodness-of-fit, or if the one-way CFA model is the worst fitting data among the competing models, it indicates that the CMB is not serious [45]. Whilst this method is one of the commonly employed approaches for detecting common method bias (CMB) within academia, it suffers from limitations in its testing power. Therefore, combining it with methods possessing relatively higher testing efficacy—such as the Unmeasured Common Method Control (ULMC) approach or the CFA label variable method—may prove a sound strategy. Any testing method merely provides evidence regarding the severity of CMB; only when CMB is judged to be severe can the influence of common method variance (CMV) be considered substantial [46].The results of this study using the CFA method showed that the model fit indices were as follows: χ²/df = 13.980, CFI = 0.516, TLI = 0.492, NFI = 0.498 (all < 0.900), and RMSEA = 0.143 (> 0.080). The model fit was poor, suggesting that CMB was not severe in this study. However, reporting bias remains a limitation of this research.

### General information of nurses

The age of 634 nurses in this study was 23～ 59 (32.86 ± 5.689) years, and other information is given in Table 1 .

**Table 1:** Sample distribution

Demographic variables	Category	Frequency (percentage/%)
Age	≤25 years	49 (7.7)
	26–30 years old	215 (33.9)
	31–35 years old	159 (25.1)
	36–40 years old	160 (25.2)
	>40 years old	51 (8.0)
Sex	Male	24 (3.8)
	Female	610 (96.2)
Nursing age	≤3 years	68 (10.7)
	4–6 years	131 (20.7)
	7–9 years	127 (20)
	10–15 years	198 (31.2)
	≥16 years	110(17.4)
Literacy	College and below	11 (1.7)
	Undergraduate	609 (96.1)
	Master and above	14 (2.2)
Marital Status	Married	441 (69.6)
	Unmarried	182 (28.7)
	Divorced	9 (1.4)
	Widowed	2 (0.3)
Title	Nurse	41 (6.5)
	Nurse	253 (39.9)
	Nurse-in-charge	315 (49.7)
	Associate chief nurse and above	25 (3.9)
Department	Internal medicine	363 (57.3)
	Surgery	98 (15.5)
	Obstetrics and Gynaecology	47 (7.4)
	Paediatrics	16 (2.5)
	Emergency Medicine	9 (1.4)
	Outpatient Department	8 (1.3)
	Department of Critical Care Medicine	77 (12.1)
	Others	16 (2.5)
Length of time working with nurse manager	≤3 years	158 (24.9)
	4–6 years	137 (21.6)
	7–9 years	144 (22.7)
	10–15 years	134 (21.1)
	≥16 years	61 (9.6)

### Reliability and validity tests

**Fig. 2:**
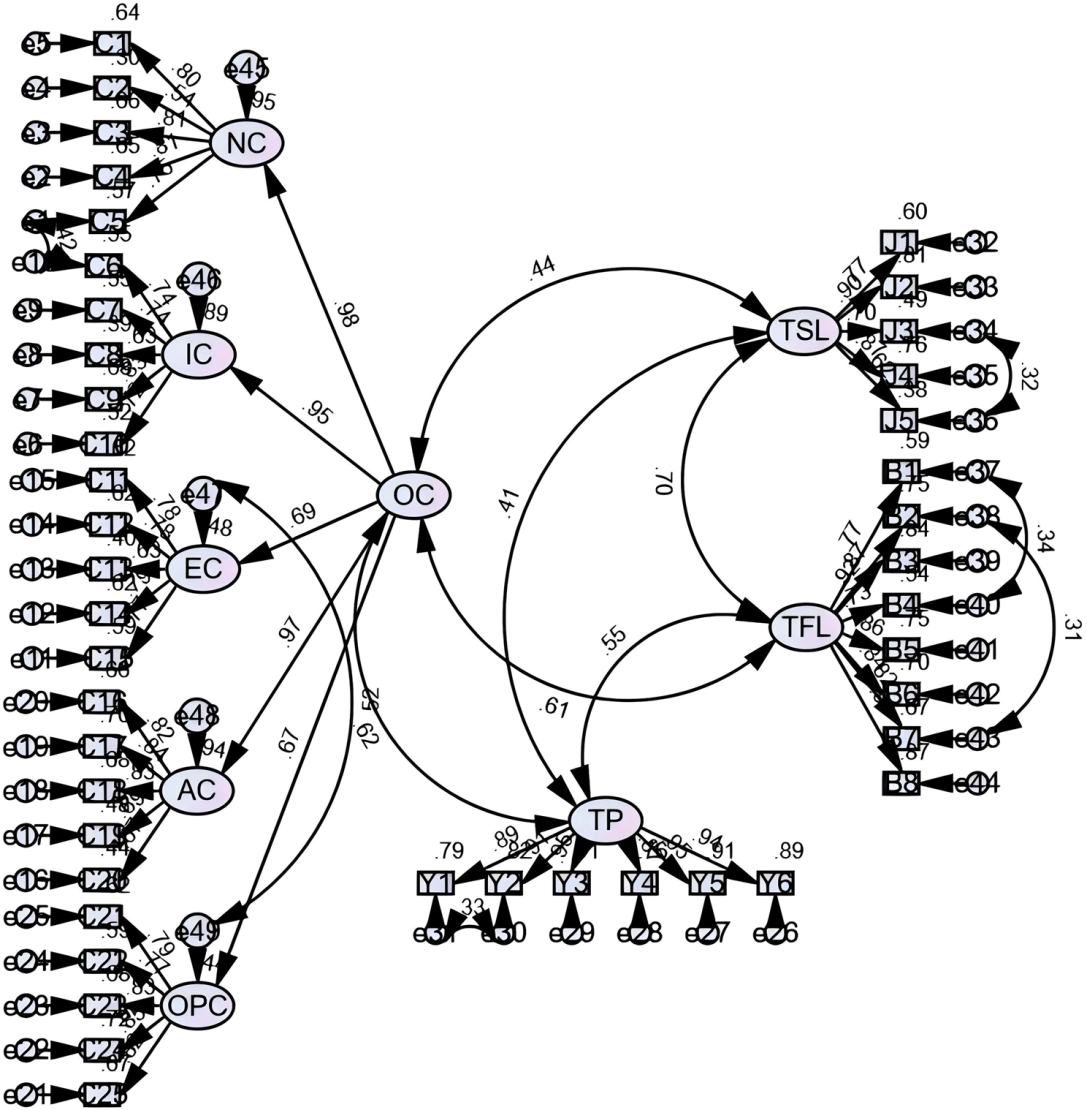
The CFA measurement model figure

We assessed the psychometric properties (reliability and validity) of the ambidextrous leadership, organizational commitment, and team performance scales. The results showed that the Cronbach’s α values were all over 0.8, indicating high reliability of the data. All scales employed in this study are validated, established instruments. Through confirmatory factor analysis (see Fig. 2 ), the model fit indices were as follows: χ²/df = 2.708, RMSEA = 0.052 (< 0.080), SRMR = 0.049 (< 0.05), CFI = 0.937, NFI = 0.905, TLI = 0.933 (all > 0.900), indicating good model fit .As shown in Table 2, the factor loadings of all observed variables corresponding to their respective latent variables exceeded 0.5, indicating that the items under each scale dimension were highly representative. Furthermore, the average variance extracted (AVE) for all latent variables was greater than 0.5, and the composite reliability (CR) for each exceeded 0.8, supporting satisfactory convergent validity. Table 3 reveals that all variables were significantly correlated (*p* < 0.001) while the absolute values of the correlation coefficients remained lower than the square root of the corresponding AVE. These results suggest that the dimensions are meaningfully related yet sufficiently distinct, thereby supporting discriminant validity. For organizational commitment as a second-order factor, convergent validity was assessed via second-order factor loadings and AVE. The AVE calculated from these loadings was 0.74, and the composite reliability reached 0.93, indicating good convergent validity for the second-order factor.The extremely high loadings (≥ 0.95) for affective commitment, normative commitment, and ideal commitment within the second-order organizational commitment construct warrant attention. This may indicate potential item redundancy or significant empirical overlap among affective, normative, and ideal commitment within the examined healthcare context. While suggesting a highly cohesive higher-order factor, it also implies that unique variance within these subdimensions may be constrained. This empirical finding, while not negating the theoretical distinctions between these dimensions, suggests that future research could optimize their operational design to more accurately capture unique variance. No items were omitted.

**Table 2:** Convergent validity of study variables

Path			Factor loadings	AVE	CR
NC	<---	OC	0.977	0.74	0.93
IC	<---	OC	0.946		
EC	<---	OC	0.690		
AC	<---	OC	0.969		
OPC	<---	OC	0.666		
C5	<---	NC	0.754	0.56	0.86
C4	<---	NC	0.808		
C3	<---	NC	0.811		
C2	<---	NC	0.544		
C1	<---	NC	0.802		
C10	<---	IC	0.720	0.54	0.85
C9	<---	IC	0.827		
C8	<---	IC	0.627		
C7	<---	IC	0.745		
C6	<---	IC	0.740		
C15	<---	EC	0.765	0.57	0.87
C14	<---	EC	0.786		
C13	<---	EC	0.631		
C12	<---	EC	0.785		
C11	<---	EC	0.785		
C20	<---	AC	0.666	0.60	0.88
C19	<---	AC	0.693		
C18	<---	AC	0.826		
C17	<---	AC	0.839		
C16	<---	AC	0.815		
C25	<---	OPC	0.822	0.66	0.91
C24	<---	OPC	0.848		
C23	<---	OPC	0.826		
C22	<---	OPC	0.770		
C21	<---	OPC	0.790		
Y6	<---	TP	0.942	0.85	0.97
Y5	<---	TP	0.952		
Y4	<---	TP	0.870		
Y3	<---	TP	0.953		
Y2	<---	TP	0.906		
Y1	<---	TP	0.888		
J1	<---	TSL	0.772	0.61	0.88
J2	<---	TSL	0.898		
J3	<---	TSL	0.698		
J4	<---	TSL	0.874		
J5	<---	TSL	0.619		
B1	<---	TFL	0.769	0.72	0.95
B2	<---	TFL	0.869		
B3	<---	TFL	0.916		
B4	<---	TFL	0.734		
B5	<---	TFL	0.864		
B6	<---	TFL	0.838		
B7	<---	TFL	0.820		
B8	<---	TFL	0.935		

AVE, average variance extracted; CR, composite reliability; TFL, Transformational leadership; TP, Team performance; TSL, Transactional leadership; OC, Organizational commitment; NC, Normative commitment; IC, Ideal commitment; EC, Economic commitment; AC, Affective commitment; OPC, Opportunity commitment.

**Table 3:** Discriminant validity of study variables

Variable	1	2	3	4
1 Transformational Leadership	**0.85**			
2Transactional Leadership	0.70^***^	**0.78**		
3 Organizational commitment	0.61^***^	0.44^***^	**0.86**	
4Team performance	0.55^***^	0.41^***^	0.63^***^	**0.92**

Notes ***p < 0.001; Bolded values on the diagonals represent the square root of AVE

### Status of two leadership styles, organizational commitment, and team performance of nurse leaders

According to the results of the descriptive analysis, among the leadership styles perceived by the subjects, transformational scores were higher than transactional scores; the total score of nurse team performance was (25.50 ± 3.65); the average scores of each dimension of nurses’ organizational commitment, from highest to lowest, were: normative commitment (4.00 ± 0.61), ideal commitment (3.94 ± 0.60), affective commitment (3.79 ± 0.70), economic commitment (3.46 ± 0.82), and opportunity commitment (3.30 ± 0.89).See Table 4 .

**Table 4:** Scores for the two leadership styles, organizational commitment and team performance ($\:\stackrel{\mathrm{-}}{\mathrm{x}}$ ± s, *n* = 634)

Variable	Number of entries (*n*)	Total score (points)	Entry score (points)
Transformational Leadership	8	31.49 ± 6.37	3.94 ± 0.80
Transactional Leadership	5	19.06 ± 4.05	3.81 ± 0.81
Organizational commitment	25	92.47 ± 15.35	3.70 ± 0.61
Normative ommitment	5	20.00 ± 3.03	4.00 ± 0.61
Ideal commitment	5	19.69 ± 3.00	3.94 ± 0.60
Economic commitment	5	17.32 ± 4.10	3.46 ± 0.82
Affective commitment	5	18.97 ± 3.49	3.79 ± 0.70
Opportunity commitment	5	16.50 ± 4.47	3.30 ± 0.89
Team performance	6	25.50 ± 3.65	4.25 ± 0.61

### Correlation between two leadership styles of nurse managers and nurses’ organizational commitment and team performance

The results of the correlation analysis indicate that both transformational and transactional leadership styles are positively correlated with organizational commitment and team performance, and the correlations are significant at the 0.05 level.Notably, the correlations of transformational leadership with organizational commitment and team performance are stronger than those of transactional leadership. There is a significant positive correlation between organizational commitment and team performance (*r* = 0.527, *P* < 0.01), indicating a moderate level of association. Empirical evidence also shows a significant positive correlation between transformational and transactional leadership styles. This finding provides empirical support for the ambidextrous leadership model, suggesting that leaders can simultaneously implement high levels of both leadership styles.See Table 5 .

**Table 5:** Correlation analysis of two leadership styles, organizational commitment and team performance (r-value)

AL	AL	TFL	TSL	OC	TP
	1.000				
TFL	0.852**	1.000			
TSL	0.908**	0.588**	1.000		
OC	0.455**	0.530**	0.298**	1.000	
TP	0.487**	0.542**	0.343**	0.527**	1.000

Notes AL, Ambidextrous leadership; TFL, Transformational leadership; TP, Team performance; TSL, Transactional leadership; OC, Organizational commitment.**p < 0.01

### Mediating effect of organizational commitment between nurse leaders’ ambidextrous leadership style and team performance

**Fig. 3:**
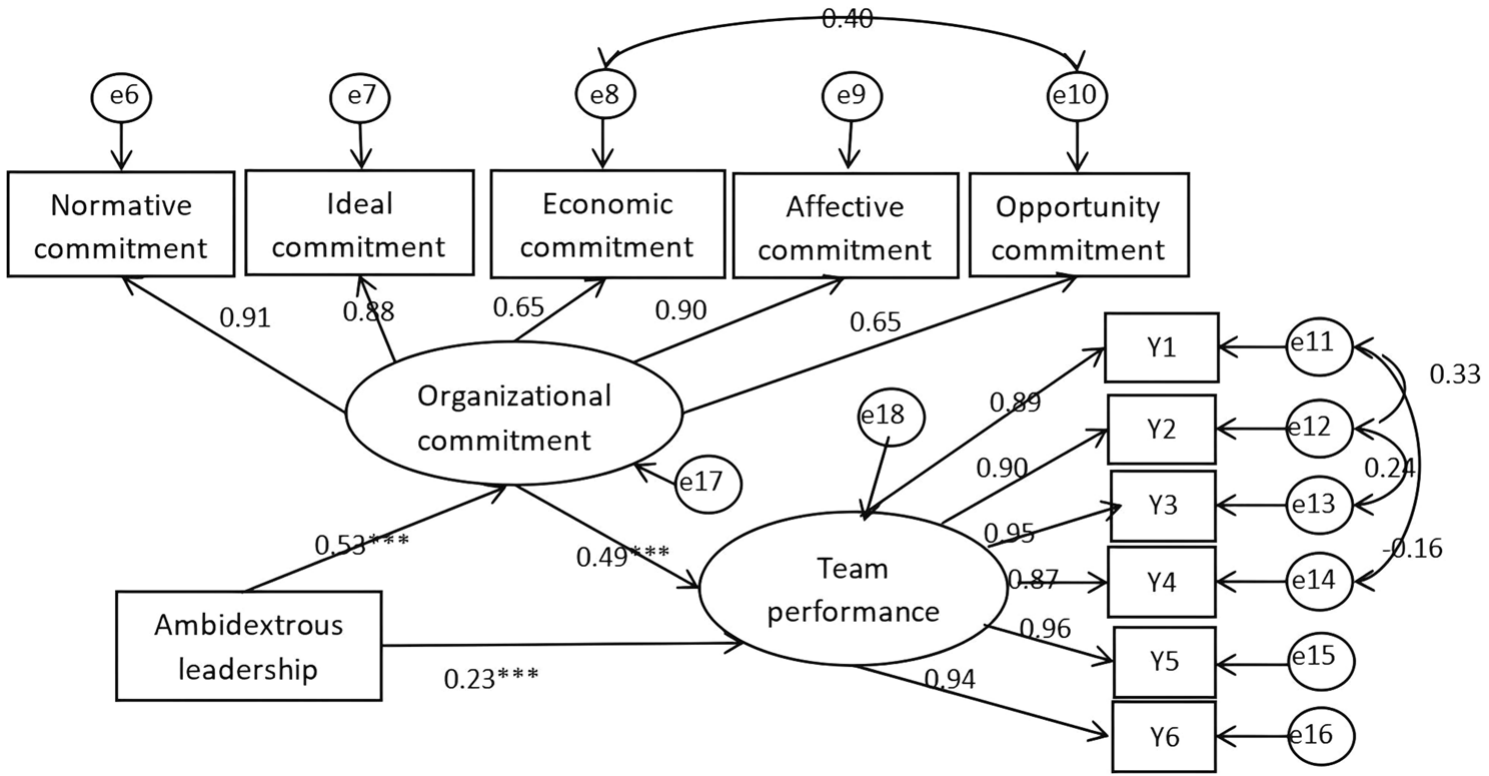
Model of mediating effect of nurses’ organizational commitment between ambidextrous leadership style and team performance. Notes***P < 0.001

A structural equation model was constructed with ambidextrous leadership as the independent variable, organizational commitment as the mediator, and team performance as the outcome variable (see Fig. 3 ). The results revealed that ambidextrous leadership had a direct positive effect on organizational commitment (β = 0.53, *p* < 0.001), supporting (Hypothesis 2) .Organizational commitment positively predicted team performance (β = 0.49, *p* < 0.001), supporting Hypothesis 3. Ambidextrous leadership also showed a direct positive effect on team performance (β = 0.23, *p* < 0.001), supporting (Hypothesis 1). See Table 6.

To validate the mediation, the bias-corrected percentile Bootstrap method was applied [47], with the number of bootstrap samples set to 5,000 and the confidence interval set at 95%. As shown in Table 7, the indirect effect was 0.027 (95% CI [0.021, 0.034], *p* < 0.001), and the direct effect was 0.024 (95% CI [0.016, 0.033], *p* < 0.001). None of the confidence intervals contained zero, and the indirect effect accounted for 52.94% of the total effect. Thus, organizational commitment played a significant partial mediating role in the relationship between ambidextrous leadership and team performance, supporting Hypothesis 4.

Post-modification model fit indices indicated good model fit: χ²/df = 3.804, CFI = 0.983, TLI = 0.983, NFI = 0.977 (all > 0.900), RMSEA = 0.067 (< 0.080), SRMR = 0.038 (< 0.05).

**Table 6:** Mediated effects path tests

Path	Standardised coefficient (β)	CR	*P*
AL-TP	0.23	6.146	0.001
AL -OC	0.53	14.617	0.001
OC-TP	0.49	11.799	0.001

Notes AL, Ambidextrous leadership; TP, Team performance; OC, organizational commitment

**Table 7:** Mediated, direct, and total effect analyses of nurses’ organizational commitment

Path	Effect value	SE	95% CI			Percentage of effect (%)
			Lower	Upper	*P*	
Indirect effect	0.027	0.003	0.021	0.034	<0.001	52.94
Direct effect	0.024	0.004	0.016	0.033	0.001	47.06
Total effect	0.051	0.004	0.042	0.060	0.001	

## Discussion

The study found that ambidextrous leadership was positively correlated with subordinates’ organizational commitment and perceived team performance. Using structural equation modeling, the impact of ambidextrous leadership and organizational commitment on perceived team performance was examined, and all proposed hypotheses were supported.

### Clinical nurses’ perceived leadership styles are predominantly transformational, followed by transactional

Regarding the scores of each leadership style within ambidextrous leadership, clinical nurses in the sampled hospital predominantly perceived their leaders’ behavior as transformational. This result is consistent with findings from existing studies [48]. Transformational nurse leaders are typically characterized by charismatic qualities such as visionary insight, motivational capability, intellectual stimulation, individualized consideration, ethical exemplification, change facilitation, strong communication skills, and adaptability. They tend to emphasize dedication, focus on collective interests, and foster an atmosphere of trust within the team, practices that help enhance overall performance [49]. This aligns with the higher-level needs in Maslow’s hierarchy of needs. In contrast, nurse leaders who adopt a transactional style focus on meeting employees’ basic needs through material incentives and clear exchanges. This corresponds to the lower-level needs in Maslow’s hierarchy of needs.

Such managers prioritize procedural compliance and accuracy, motivating nursing staff by promptly correcting deviations to achieve predefined performance objectives.

By employing integrated and dialectical thinking to balance these complementary leadership approaches, nurse managers can simultaneously promote professional potential development while stimulating exploratory and innovative capacities among nursing staff [50]. The complexity of healthcare environments places high demands on the management capabilities and adaptability of head nurses. Using different behavioral strategies and management methods for different nurses and tasks is not only a necessary response to the environment but also a reflection of nursing management competence.

### Organizational commitment, team performance scores at a high level

The results of this study indicate that the organizational commitment score (92.47 ± 15.35) is at a moderate to high level, suggesting that nurses exhibit a strong organizational commitment to the hospital. This finding is consistent with the results of Li Na et al. [51]. However, scores for opportunity commitment and economic commitment were relatively low, with opportunity commitment being the lowest. This suggests that there is considerable room for improvement in these areas within the hospital. The low levels of economic and opportunity commitment imply that the costs of nursing staff leaving their positions are minimal, and nurses have a high level of mobility, facing fewer constraints. The survey results show that 98.3% of nursing staff at the target hospital hold at least a bachelor’s degree, and the higher the educational level, the greater the employment opportunities. At the same time, the current salary does not provide strong enough incentives for nursing staff. It is recommended that nursing administrators focus on cultivating nurses’ opportunity-based organizational commitment [52], enhance career planning for nurses, and help them achieve both personal and societal value, further increasing their overall organizational commitment and providing a stable nursing workforce to support the hospital’s development.

The performance score of the target nursing team was (25.50 ± 3.65), also at a moderate to high level, indicating generally high performance. This can be attributed not only to the leadership style of the head nurses but also to the performance reforms actively promoted by the hospital in recent years. In accordance with the “Healthy China 2030” Planning Outline and the State Council’s Guidelines on Strengthening Performance Evaluation in Public Tertiary Hospitals (Guo Ban Fa [2019] No. 4), tertiary public hospitals have established a modern management system to promote high-quality development and improve the overall quality of healthcare services. As a key component, performance has always been a major concern for hospital administrators and researchers. Key performance indicators—such as the coverage rate of high-quality nursing wards, single-disease quality control, and healthcare worker satisfaction—are directly linked to the performance of the nursing team [53]. The high level of team performance achieved by the target hospital’s nursing team can be attributed not only to its policy-driven approach and scientifically sound performance evaluation system but also to the positive work environment fostered by the head nurses’ ambidextrous leadership in daily management.

### The ambidextrous leadership style of the head nurse is positively correlated with team performance

The results of the correlation analysis show a significant positive correlation between head nurses’ ambidextrous leadership and team performance. Path analysis using structural equation modeling revealed that ambidextrous leadership has a significant positive predictive effect on team performance (β = 0.23, *P* < 0.001), indicating that the synergistic effect of ambidextrous leadership directly contributes to the enhancement of team performance. The analysis suggests that, compared to a single leadership style, head nurses with an ambidextrous leadership style are able to adapt their leadership behaviors to the complex situations encountered in daily hospital management. They flexibly address contradictions in the workplace and, in addition to providing the necessary material rewards to goal-oriented employees, implement personalized care strategies and motivational actions. This approach helps establish a long-term system of protection, care, and support for nursing staff [54]. By leveraging leaders’ charisma and inspirational influence, it motivates employees to achieve higher levels of engagement, thereby enabling teams to sustain performance outcomes in their work. In this process, the exchange relationship between leaders and subordinates also begins to expand from simple economic exchange to a complex exchange encompassing social exchange. This study provides empirical evidence that ambidextrous leadership exerts a positive influence on team performance.

### Organizational commitment plays a partial mediating role between nurse leaders’ ambidextrous leadership style and team performance

The results indicate that organizational commitment partially mediates the relationship between ambidextrous leadership and team performance. Partial mediation effects indicate that while ambidextrous leadership can directly enhance team performance, the majority of its impact is achieved through strengthening nurses’ organizational commitment.Existing studies show that ambidextrous leadership has a positive effect on organizational commitment, which serves as a mediator [32]. Organizational commitment has been proven to have a positive impact on both team performance and individual performance.According to Leader-Member Exchange (LMX) theory, high-quality exchange relationships not only enhance employees’ organizational commitment but also positively impact personal effectiveness and leadership effectiveness, ultimately affecting overall team performance. Head nurses possessing ambidextrous leadership capabilities demonstrate situational adaptability, facilitating the formation of high-quality exchange relationships with subordinates. When leader-member relationships progress to the socialisation stage, establishing stable trust between parties further strengthens subordinates’ organizational commitment.The study of Wu Shijian et al. [55] proved that high affective commitment of employees will make them identify more with the organizational goals, willing to consider problems from the organization’s point of view, and make altruistic behaviours.Therefore, nursing managers may leverage organizational commitment as a starting point, harnessing the strengths of ambidextrous leadership to enhance staff organizational commitment levels.

### Implications

Grounded in leader-member exchange (LMX) theory, this study situates leaders (head nurses) and members (nurses) within the organizational context of nursing teams to systematically examine how ambidextrous leadership influences nursing team performance through organizational commitment. The research not only delineates the pathway through which ambidextrous leadership affects team performance but also offers evidence-based insights for hospitals to enhance the effectiveness of nursing teams, contributing to both theoretical and practical domains.

At the theoretical level, this study introduces organizational commitment as a mediating variable to elucidate the underlying mechanism through which ambidextrous leadership impacts team performance. By constructing and validating a mediation model of “ambidextrous leadership → organizational commitment → team performance,” it extends the application of ambidextrous leadership theory into nursing management and broadens the perspective on the relationship between leadership and team performance. Furthermore, it provides new empirical support for LMX theory within healthcare organizational settings.

On the practical level, this study provides the following insights for nursing team management. First, nursing administrators should shift their management mindset and actively recognize and cultivate their own ambidextrous thinking and leadership abilities to enhance subordinates’ work motivation. At the same time, they should pay more attention to enhancing subordinates’ organizational commitment and implement measures to elevate employees’ commitment levels. Second, hospital administrators can implement training programs aimed at enhancing ambidextrous leadership cognitive thinking, incorporating them into the guidance and supervision systems, and focusing on improving head nurses’ ability to perceive changes in the external environment and understand internal team needs. By providing institutional and environmental support, nursing staff’s organizational commitment can be strengthened, thereby driving the nursing team toward high performance and contributing to the continuous improvement of the hospital’s overall service quality. 

### Inadequacies of this study

Firstly, owing to the limited time and funding available to the researchers, this study employed convenience sampling to select eligible clinical nurses from a single tertiary hospital. This approach may have somewhat restricted the representativeness of the sample.The proportion of female participants in this study far exceeded that of males, resulting in a significant gender imbalance. The underrepresentation of male participants may have influenced the formulation of certain responses or viewpoints, thereby potentially affecting the generalisability of the research findings.In the future, a more scientific and rigorous approach could be used to further validate the findings in hospitals of all levels in Anhui Province or even in other provinces in China. Secondly, this study is a cross-sectional investigation, a methodology that can only provide correlational information without establishing causality. Consequently, it cannot determine whether managers exhibiting strong ambidextrous leadership lead to higher organizational commitment or performance outcomes. To explore the relationship between these three factors in greater depth, future research could adopt a longitudinal design to uncover potential causal associations.Finally, similar to prior studies, this research relied on self-reported questionnaires. Although common method bias was statistically examined, the results may still be subject to the Dunning-Kruger effect [56]. Future studies could adopt multisource ratings to reduce such bias.

## Conclusion

In conclusion, our study found that perceived organizational commitment plays a significant partial mediating role in the relationship between head nurses’ ambidextrous leadership and team performance. Future research could explore this further using longitudinal tracking and other dynamic research methods to capture the behavioral transformation process of ambidextrous leadership in medical practice. Intervention studies could also be conducted to validate the effectiveness of ambidextrous leadership development programs. Given the gender imbalance in the nursing field, future studies may focus on male nurses’ perceptions and views regarding their leaders’ leadership styles. Overall, the findings of this study will provide valuable guidance for nursing management practices in public hospitals and play a positive role in enhancing the quality of healthcare services.

## Data Availability

The data in this study can be obtained from the corresponding author on reasonable request.
